# Role of Adjuvant Radiotherapy in Sweat Gland Carcinoma: A Case Report and Literature Review

**DOI:** 10.7759/cureus.77628

**Published:** 2025-01-18

**Authors:** Arit Bhattacharjee, Debarshi Lahiri, Ranti Ghosh, Debanjan Chakraborty

**Affiliations:** 1 Radiation Oncology, Chittaranjan National Cancer Institute, Kolkata, IND

**Keywords:** adjuvant radiation therapy, clinical case report, immunohistochemistry, sweat gland carcinoma, wide local excision

## Abstract

Sweat gland carcinoma (SGC) is a rare type of malignant adnexal tumours of the skin (MATS). It has potential for local infiltration, regional nodal involvement, and distant metastasis. Wide local excision with regional lymph nodal dissection is the mainstay of treatment and there are no consensus guidelines for adjuvant treatment. We present a case report of a gentleman, suffering from SGC in the left lumbar region with axillary nodal metastasis, presenting with a longstanding skin nodule, with recent spurt in growth and ulceration over it. He was treated with wide local excision of the lesion and left axillary nodal dissection. Postoperative histopathology and immunohistochemistry confirmed the diagnosis of SGC. He received adjuvant radiotherapy to primary and nodal sites due to the presence of high-risk features. Diagnosis of SGC is very challenging due to clinical variation, nonspecific immunohistochemistry profile, and histopathological surprises. Due to the paucity of relevant literature, the role of adjuvant radiotherapy is still not established, but in view of the aggressive behaviour of SGC, radiotherapy significantly improves local and regional control, particularly in the presence of high-risk pathological features. Further studies in the domain of adjuvant treatment of SGC is needed to improve disease outcomes optimally.

## Introduction

Sweat gland carcinoma (SGC) is a very rare malignant adnexal tumour of the skin (MATS), comprising less than 0.01% of cutaneous cancers [1]. Arising from the pilosebaceous unit and eccrine and apocrine sweat glands, SGC usually occurs in the fifth to seventh decades of life, with similar gender preponderance. The most common sites are the head and neck region, axilla, inframammary region, perineum, and lower extremities [2]. Starting as an asymptomatic nodule, it often shows aggressive growth kinetics later - also local invasiveness, lymphatic involvement, and even distant metastases [3]. Clinically, variable presentation, accidental discovery during histopathological examination (HPE), and furthermore complex immunohistochemistry (IHC) profiling makes the diagnosis challenging. The paucity of relevant literature makes the management of SGC even more difficult. Surgical excision with regional lymph node dissection (LND) is standard treatment, but the role of adjuvant treatment is not very clear due to limited evidence.

Here, we present a case of SGC of the left lumbar region revealing challenges in diagnosis and management, emphasizing the role of adjuvant treatment in tumors having a higher risk of locoregional relapse.

## Case presentation

**Figure 1:**
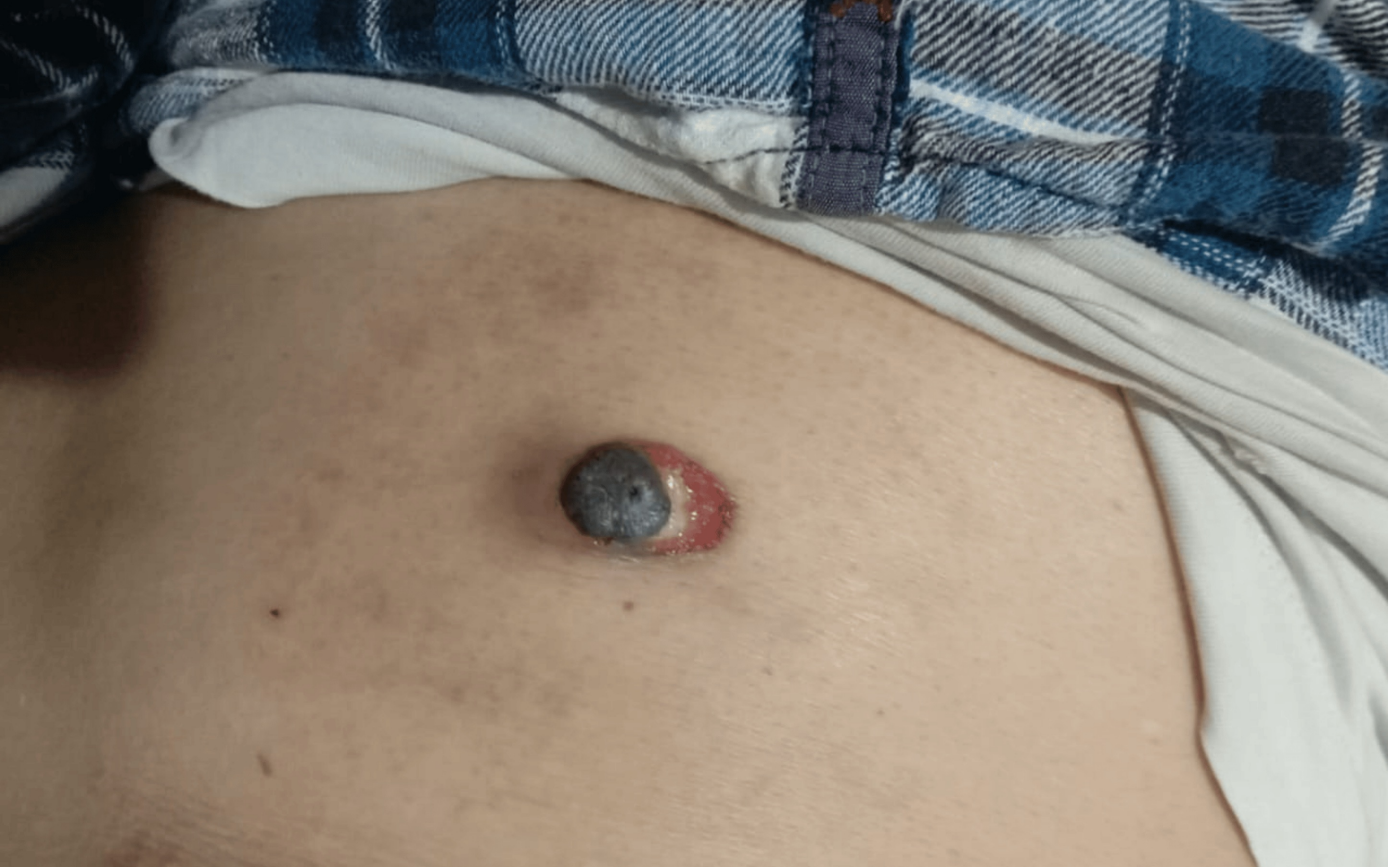
Clinical photograph of the skin lesion

A 69-year-old gentleman with hypertension and ischemic heart disease, in the month of June 2023, presented with a persistent asymptomatic nodule, in the left lumbar region, which was gradually progressing in size since the last three months. Clinically, the lesion was 4 x 3 cm in size, hard, painless, and ulcerated and had ill-defined margins (Figure 1), suspicious for a cutaneous malignancy.

After routine lab investigations, a positron emission tomography/computed tomography (PET-CT) scan done on September, 2023, showed FDG avid cutaneous thickening in left lumbar region approximately 3.1 cm anteroposteriorly, 1.7 cm thick, SUV max 12.1, with an 8 x 10 mm sized hypermetabolic left axillary lymphadenopathy, SUV max 8.9, which stages the tumor to be cT2N1M0 (Stage III, as per AJCC TNM staging for non-melanoma skin cancers). FNAC from both primary tumor and node showed squamous cell carcinoma (SCC).

**Figure 2:**
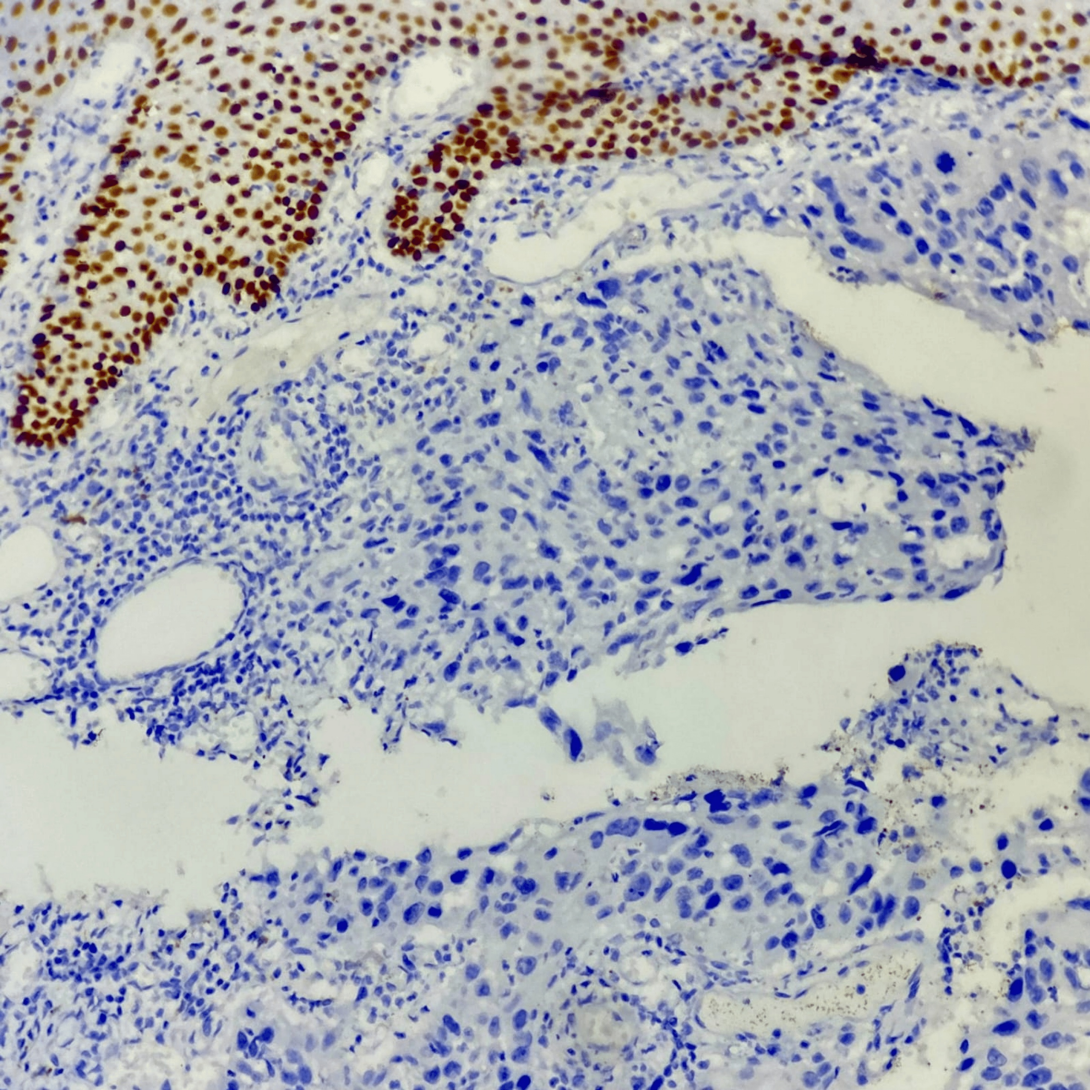
Immunohistochemistry slide showing p63 positivity

The patient underwent wide local excision (WLE) of the lesion and rotational fasciocutaneous flap reconstruction, with ipsilateral axillary node dissection in the last week of September 2023. Surgical HPE showed SCC, tumor size 3 x 3.2 cm, Grade 3, with lymphovascular invasion (LVI) and perineural invasion (PNI) present. Overlying ulcerated skin and subcutaneous tissue infiltration up to 1.3 cm depth was noted. All surgical margins were free of tumour. Four out of 23 axillary nodes (4/23) were involved by carcinoma without any extra-nodal extension (ENE). IHC showed positivity for CK7, CK5/6, and P63 (Figure 2); negative for CK20, P40, and Ki67 5% reported as MATS consistent with SGC.

We discussed the case in the Multidisciplinary Tumour Board on December 2023 due to a surprising change in histopathology. Another HPR review confirmed the diagnosis of SGC. In view of the high-grade, positive LVI and PNI, extensive lymph nodal involvement, and depth of invasion >1 cm, the patient was treated with adjuvant radiotherapy to both the primary site and axilla, to reduce the risk of the locoregional relapse.

He received external beam radiation (EBRT), starting from the first week of January 2024 till the third week of February 2024, to a dose of 60 Gy in 30 fractions to the primary tumor bed with adequate margins, for including subclinical microscopic disease (tumor bed identified by scar localization during planning CT by radio-opaque material on skin scar and pre-surgical images) using electrons of 10 MeV energy, prescribed at depth of 3 cm for optimal coverage, and a 1 cm bolus to ensure adequate skin doses. Left axillary nodes of level I-III were irradiated, to a dose of 50 Gy in 25 fractions using volumetric modulated arc therapy (VMAT), to minimize normal tissue toxicities, ensuring limited doses as per tolerance of critical organs at risk - left lung, heart, left humeral head, neurovascular bundle, and spinal cord.

EBRT was completed in February 2024 over a period of six weeks with only Grade I acute dermatitis. The patient is still alive and disease free nine months since the RT conclusion, pursuing clinical follow up every three to four months now, with good functional and psychosocial quality of life (QoL).

## Discussion

Due to the limited literature, variable presentation, difficult pathological classification, diagnosis, and treatment, there is no consensus guideline for management of SGC.

After the pathological classification of SGC by Berg in 1968 [4], the WHO 2018 classification divided SGC into 10 subtypes: adnexal adenocarcinoma, syringocystadenocarcinoma papilliferum, clear cell hidradenocarcinoma, porocarcinoma, mucinous carcinoma, and others [5]. Traditionally, SGCs are divided into apocrine and eccrine, sometimes having both features. There is insufficient evidence to suggest any impact of tissue origin or WHO classification on management [6-7]. Occurring in the fifth to seventh decades of life, with similar gender preponderance, most usual sites of SGC are the head and neck, followed by the lower limbs. The pattern of relapse is mostly regional nodes, followed by the lung [2].

In our patient, SGC was present on the left lumbar region, an uncommon site, and it spread to the axillary nodes without any abdominal-pelvic nodal disease. In absence of obvious histopathology, it is very difficult to differentiate from benign or other analogous malignant counterparts, so very often, it can surprise clinicians. IHC with expressions of P53, P63, and CK7 is very suggestive of SGC [8]. Similarly, in our patient, initially, the clinicoradiological, FNAC, and histopathology of the operative specimen suggests SCC of the skin, whereas the aforesaid IHC results changed the diagnosis to SGC.

The recommended treatment of SGC is full-thickness WLE with at least 1-2 cm margin, preferably by Mohs micrographic surgery, which shows the lowest rate of local relapse in aggressive skin malignancies [9-10]. Upfront 50% risk of lymph nodal involvement dictates the role of elective LND in SGC, even in clinically node negative status, keeping in mind the role of sentinel lymph node mapping [11]. Although the literature is scarce, the role of radiotherapy in reducing the rates of locoregional relapse (which are higher otherwise, up to 50%) is of paramount importance [12]. Pathological features such as dermal LVI, PNI, deep infiltration, positive resection margins, anaplasia, and ENE may identify a higher risk of recurrence, and adjuvant EBRT is clinically meaningful there, up to 60-70 Gy doses, with good local control, supported by recent evidences [6,13]. Definitive EBRT doses up to 60-70 Gy radiation in the head and neck SGC improved local control greatly [14].

In our patient, as HPE showed four high-risk features (positive LVSI, PNI, high grade, and deep tissue infiltration), adjuvant RT was delivered up to 60 Gy dose to the primary disease and 50 Gy to the axillary nodes. No dose escalation was done due to clear margins and no ENE. The usage of electrons helped to achieve complete target coverage, dose distribution, and proper reproducible set-up during treatment. The VMAT technique for the axilla maximized normal tissue sparing.

Although robust evidences are unavailable, multiple chemotherapeutic agents such as 5-fluorouracil, doxorubicin, cyclophosphamide, paclitaxel, mitomycin C, vincristine, and cisplatin had been used in SGC, mostly with short-term response and potential toxicity. Another case report showed a patient of metastatic SGC, treated with doxorubicin, mitomycin C, vincristine, and cisplatin, who achieved complete response, sustaining for 1.5 years. Docetaxel has also been used with a good response rate [15].

## Conclusions

SGC is a rare aggressive cancer, belonging to the malignant skin adnexal tumour group. Multifaceted clinical presentation and pathological complexity makes the diagnosis difficult, making IHC so crucial for accurate characterisation. Mohs micrographic surgery followed by risk-adapted adjuvant EBRT is the preferred treatment approach for optimal outcome in localized SGC. No standard guideline is available for the use of chemotherapy and Immunotherapy. Furthermore, prospective studies are necessary to develop consensus treatment guideline.

## References

[1] Chadha S, Kumar R, Singhal S, Ruhela S (2021). Primary cutaneous apocrine carcinoma: case report and literature review. Indian J Pathol Microbiol.

[2] Salih AM, Kakamad FH, Baba HO (2017). Porocarcinoma; presentation and management, a meta-analysis of 453 cases. Ann Med Surg (Lond).

[3] Chintamani Chintamani, Sharma R, Badran R, Singhal V, Saxena S, Bansal A (2003). Metastatic sweat gland adenocarcinoma: a clinico-pathological dilemma. World J Surg Oncol.

[4] Gates O, Warren S, Warvi WN (1943). Tumors of sweat glands. Am J Pathol.

[5] Segal A, Segal N, Gal A, Tumuluri K (2010). Mucinous sweat gland adenocarcinoma of the eyelid - current knowledge of a rare tumor. Orbit.

[6] Dai Y, Feng J, Zhou X (2023). Case report: case report and literature review: treatment of sweat gland carcinoma. Front Oncol.

[7] Snow S, Madjar DD, Hardy S (2001). Microcystic adnexal carcinoma: report of 13 cases and review of the literature. Dermatol Surg.

[8] Soni A, Bansal N, Kaushal V, Chauhan AK (2015). Current management approach to hidradenocarcinoma: a comprehensive review of the literature. Ecancermedicalscience.

[9] Wildemore JK, Lee JB, Humphreys TR (2004). Mohs surgery for malignant eccrine neoplasms. Dermatol Surg.

[10] Thomas CJ, Wood GC, Marks VJ (2007). Mohs micrographic surgery in the treatment of rare aggressive cutaneous tumors: the Geisinger experience. Dermatol Surg.

[11] Delgado R, Kraus D, Coit DG, Busam KJ (2003). Sentinel lymph node analysis in patients with sweat gland carcinoma. Cancer.

[12] Harari P, Shimm D, Bangert J, Cassady J (1990). The role of radiotherapy in the treatment of malignant sweat gland neoplasms. Cancer.

[13] Zhang Z, Yin S, Xu Z, Wang S (2023). Sweat gland carcinoma of the head and neck: case report and literature review. Ear Nose Throat J.

[14] Sasamura K, Matsubara D, Kojima M, Yuasa-Nakagawa K, Toda K, Miura K, Yoshimura R (2019). Intensity modulated radiation therapy for syringomatous carcinoma of the face: a case report. Adv Radiat Oncol.

[15] Piedbois P, Breau JL, Morere JF, Israel L (1987). Sweat gland carcinoma with bone and visceral metastases. prolonged complete remission lasting 16 months as a result of chemotherapy. Cancer.

